# Genomic analysis of *Ralstonia pickettii* reveals the genetic features for potential pathogenicity and adaptive evolution in drinking water

**DOI:** 10.3389/fmicb.2023.1272636

**Published:** 2024-02-02

**Authors:** Chao Yuan, Tianfeng An, Xinlong Li, Jiao Zou, Zhan Lin, Jiale Gu, Ruixia Hu, Zhongze Fang

**Affiliations:** ^1^Department of Toxicology and Sanitary Chemistry, School of Public Health, Tianjin Medical University, Tianjin, China; ^2^Tianjin Key Laboratory of Environment, Nutrition and Public Health, Tianjin Medical University, Tianjin, China; ^3^Center for International Collaborative Research on Environment, Nutrition and Public Health, School of Public Health, Tianjin Medical University, Tianjin, China; ^4^School of Public Health, Tianjin Medical University, Tianjin, China

**Keywords:** *Ralstonia pickettii*, pan-genome, core-genome, pathogenicity, adaptive evolution

## Abstract

*Ralstonia pickettii*, the most critical clinical pathogen of the genus *Ralstonia*, has been identified as a causative agent of numerous harmful infections. Additionally, *Ralstonia pickettii* demonstrates adaptability to extreme environmental conditions, such as those found in drinking water. In this study, we conducted a comprehensive genomic analysis to investigate the genomic characteristics related to potential pathogenicity and adaptive evolution in drinking water environments of *Ralstonia pickettii*. Through phylogenetic analysis and population genetic analysis, we divided *Ralstonia pickettii* into five Groups, two of which were associated with drinking water environments. The open pan-genome with a large and flexible gene repertoire indicated a high genetic plasticity. Significant differences in functional enrichment were observed between the core- and pan-genome of different groups. Diverse mobile genetic elements (MGEs), extensive genomic rearrangements, and horizontal gene transfer (HGT) events played a crucial role in generating genetic diversity. In drinking water environments, *Ralstonia pickettii* exhibited strong adaptability, and the acquisition of specific adaptive genes was potentially facilitated by genomic islands (GIs) and HGT. Furthermore, environmental pressures drove the adaptive evolution of *Ralstonia pickettii*, leading to the accumulation of unique mutations in key genes. These mutations may have a significant impact on various physiological functions, particularly carbon metabolism and energy metabolism. The presence of virulence-related elements associated with macromolecular secretion systems, virulence factors, and antimicrobial resistance indicated the potential pathogenicity of *Ralstonia pickettii*, making it capable of causing multiple nosocomial infections. This study provides comprehensive insights into the potential pathogenicity and adaptive evolution of *Ralstonia pickettii* in drinking water environments from a genomic perspective.

## Introduction

The genus *Ralstonia*, a group of aerobic gram-negative bacteria belonging to the family Burkholderiaceae, was first described in 1995 (Yabuuchi et al., 1995). Currently, seven species have been identified: *Ralstonia insidiosa*, *Ralstonia mannitolilytica*, *Ralstonia pickettii*, *Ralstonia pseudosolanacearum*, *Ralstonia solanacearum*, and *Ralstonia syzygii*, and *Ralstonia wenshanensis* (Euzéby, 1991). *Ralstonia* species have increasingly been recognized as emerging nosocomial pathogens, particularly in immunocompromised patients (Ryan et al., 2006). Among the *Ralstonia* species, *Ralstonia pickettii*, previously known as *Burkholderia pickettii*, is considered the most clinically significant pathogen. While it is generally associated with low pathogenicity, occasional nosocomial outbreaks of *Ralstonia pickettii* infections have been reported (Nasir et al., 2019; Menekşe et al., 2022). *Ralstonia pickettii* could be isolated from various clinical specimens, including blood, wounds, urine, ear, nose swabs, or cerebrospinal fluid (Stelzmueller et al., 2006). Despite its low virulence, it has been implicated in causing potentially harmful infections and even death (Chen et al., 2015). However, there is limited research on the genetic diversity and potential pathogenicity of *Ralstonia pickettii* (Ryan et al., 2011).

On the other hand, the presence of *Ralstonia pickettii* in mineral solutions such as sterile saline solution, disinfectant, or other medical solutions, specifically purified water supplies, could explain its association with nosocomial outbreaks (Riley and Weaver, 1975; Ryan and Adley, 2013; Baker et al., 2022). Interestingly, *Ralstonia pickettii* has even been isolated from the potable water system of the International Space Station (PRJNA493516 from NCBI database). This highlights the adaptability of *Ralstonia pickettii* to survive in diverse environments, including drinking water, which poses a potential public health threat. Drinking water is a unique environment characterized by limited organic nutrient availability but suitable mineral concentrations for biological requirements (Liu et al., 2021). It is not an ideal condition for the survival of heterotrophic bacteria. However, *Ralstonia pickettii* has demonstrated remarkable environmental adaptability and the ability to thrive under extreme conditions, such as drinking water. This adaptability raises concerns about its potential to cause infectious diseases in future. The genetic mechanisms underlying its adaptation to drinking water environments are currently unknown.

Although *Ralstonia pickettii* is not considered a primary pathogen and is generally believed to have low virulence, its wide distribution in human-related environments and its potential to cause harmful infections emphasize the need for appropriate medical strategies and further in-depth research. Whole-genome sequencing (WGS) provides a valuable tool for exploring evolutionary relationships, genomic characteristics, and biotechnological properties at the gene level (Yuan et al., 2019; Yin et al., 2020). Genome plasticity enables bacteria to rapidly evolve and adapt to environmental variations by modulating virulence and antimicrobial resistance, in addition to environmental adaptation (Everitt et al., 2014; Yuan et al., 2020). The selective pressure on the core genome indicates adaptive evolution under specific environmental conditions. In this study, we present a comprehensive genomic analysis to investigate the genetic diversity, potential pathogenicity, and adaptive evolution of *Ralstonia pickettii* in drinking water.

## Materials and methods

### Genome data collection

All collected genomes of *Ralstonia pickettii* species were downloaded from the NCBI GenBank database. The genome completeness and contamination assessment were performed using CheckM v1.0.13 (Parks et al., 2015). A total of 76 available WGS data of *Ralstonia pickettii* were collected from NCBI and analyzed in this research. The information of these strains is presented in Supplementary Table S1.

### Phylogenetic analysis based on single-copy core-gene orthologous gene families

The orthologous groups of protein families of pan-genome were delimited using OrthoFinder v2.5.1 (Emms and Kelly, 2015) software, employing the DIAMOND algorithm (Buchfink et al., 2015) with the default parameters. Subsequently, the single-copy orthologous gene families, core-gene families, accessory-gene families, and pan-gene families were extracted from the output results of OrthoFinder. Nucleotide sequences of the single-copy orthologous gene families were extracted and then aligned using MAFFT v7.475 (Katoh and Standley, 2013). For the phylogenetic analysis of *Ralstonia pickettii*, single-nucleotide polymorphisms (SNPs) present in the single-copy orthologous gene families were utilized. The Maximum Likelihood (ML) tree was constructed using MEGA 11 software (Kumar et al., 2016) [with the General Time Reversible (GTR) model].

### Core- and pan-genome statistics analysis

Heap’s law was applied to analyze the pan-genome models in this study. The relationship between the total number of gene families (*n*, y-axis) and the increasing number of genomes (*N*, x-axis) was examined. The curve fitting with a power-law regression is based on Heaps’ law (*n* = $кN^{\gamma })\text{,}$ where N represents the number of genomes, к is a proportionality constant, and the growth exponent γ > 0 indicates an open pan-genome. This regression analysis provides insights into the expansion of the pan-genome as more genomes are included (Heaps, 1978; Tettelin et al., 2008). Additionally, the core-genome analysis was performed using regression analysis, and curve fitting of core-genome was performed using an exponential regression model (*n* = *к* exp. (*m* N ) + *Θ*), where N represents the number of genomes and к and m are proportionality constants (Bottacini et al., 2010). Descriptive statistical analysis was generated using OriginPro 9 software with exponential function model YldFert1 (core-genome) and power function model Allometric1 (pan-genome).

### Genetic population structure analysis and gene functional category

The average nucleotide identity (ANI) was calculated using JSpecies v1.2.1 software (Richter and Rosselló-Móra, 2009). Population structure analysis was conducted using RhierBAPS v1.1.3 (Cheng et al., 2013; Tonkin-Hill et al., 2018). We analyzed the functional category of the gene family based on the Cluster of Orthologous Group (COG) assignment (Galperin et al., 2015). The functional annotation of proteins was performed using eggNOG-mapper v2.1.9 with default parameters (Huerta-Cepas et al., 2019; Cantalapiedra et al., 2021). The prophages were identified using the Phage Search Tool Enhanced Release (PHASTER; Arndt et al., 2016). Genomic islands were predicted using the IslandViewer 4 database with default parameters (Bertelli et al., 2017). HGTector (Zhu et al., 2014) was employed to identify the potential horizontal genes in *Ralstonia pickettii* species with default parameters.

### Identification of virulence genes and resistance genes

To identify the virulence genes and resistance genes, protein sequences of all *Ralstonia pickettii* genomes were aligned using BLASTp against the data set from the Pathogen Host Interactions database (PHI-base 5.0; Urban et al., 2020) and Comprehensive Antibiotic Database (CARD; Jia et al., 2017) with three screening thresholds: (1) the percentage of identical < 50% or coverage < 60%; (2) the percentage of identical >50% and percentage of identical <75% and coverage > 60%; (3) the percentage of identical > 75% and coverage > 60%. All blast results in e-value cutoff of <1e − 6. These results were visualized using the R packages pheatmap v1.0.12 and Adobe Illustrator CS6.

### Identification of macromolecular systems

The detection and visualization of Macromolecular systems in *Ralstonia pickettii* species were performed using MacSyFinder (Abby et al., 2014) and TXSScan (Abby and Rocha, 2017) within Galaxy workflow^1^ with the default parameters. The T4SS and T6SS were further analyzed using SecReT4 (Liu et al., 2012) and SecReT6 (Li et al., 2015) on the default parameters, respectively.

### Comparative core-genomic and pan-genomic analysis

The comparative core-genomic and pan-genomic analysis was conducted to investigate the genetic characteristics among different groups. Increased and decreased core-gene families were extracted and annotated by the KEGG database (database resolution through Python 3.8, based on public information from the KEGG database).Two different classification methods counted results of “Gene function” and “Pathway” and shown in a bar chart. Based on gene data above, Relative Enrichment Ratio (RER) was introduced to describe the correlation between gene enrichment and pathway (Xu and Yuan, 2022). The RER was calculated using the formula: RER = (number of genes annotated in this pathway)/(total number of genes involved in this pathway) and shown in a line chart; the RER results were screened by 0.1 and 0.2 as the threshold (maximum number of peaks can be retained), and the pathway corresponding to each peak was extracted. All figures were made by GraphPad prism 9.0, R packages, and Adobe Illustrator CS6.

## Results

### Phylogenetic and genetic population analyses of *Ralstonia pickettii*

**Figure 1:**
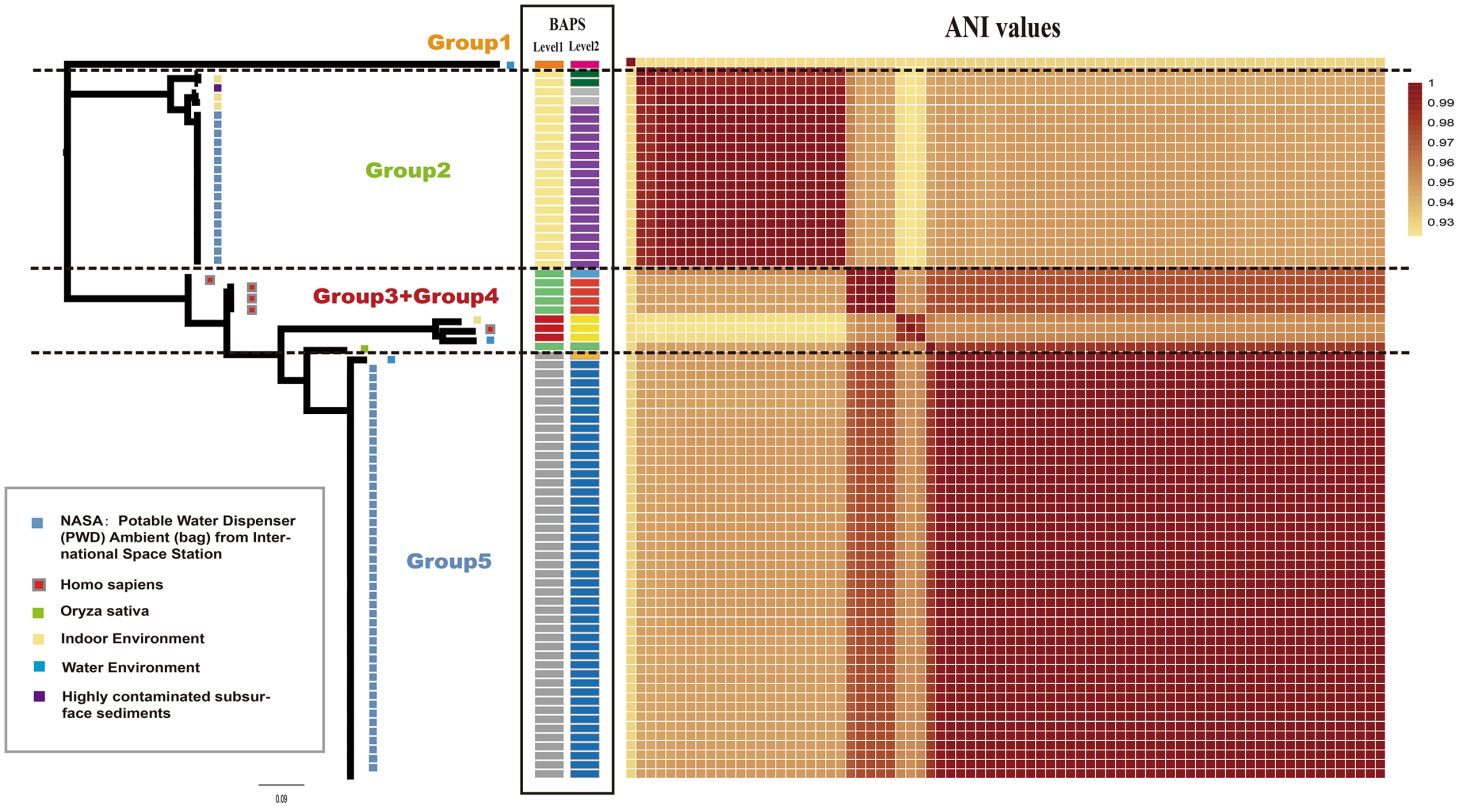
Phylogenetic relationship and isolated information of *Ralstonia pickettii*. The maximum likelihood (ML) tree was constructed using SNPs identified across 3,010 single-copy core gene families that are shared among the 76 *Ralstonia pickettii* genomes. On the right and middle of the chart, a heatmap representing the average nucleotide identities (ANI) and BAPS (two levels) is displayed. The isolated information of *Ralstonia pickettii* strains is labeled with different colors within gray frames.

A maximum-likelihood phylogenetic tree was constructed using 3,010 concatenated single-copy orthologous gene families from 76 *Ralstonia pickettii* genomes (Figure 1). To explore the genomic similarities among the strains, genetic population structure analysis was performed using Bayesian analysis of population structure (BAPS) at two levels. This analysis categorized the *Ralstonia pickettii* strains into 5 BAPS classes at Level 1 or 10 BAPS classes at Level 2. The results from the BAPS analysis were consistent with the genetic distances estimated by the average nucleotide identity (ANI) values (Figure 1). Combining the results from the phylogenetic analysis, BAPS analysis, and ANI values, the *Ralstonia pickettii* strains were divided into five groups. The majority of the strains (64/76, 84.2%) were isolated from water-related environments. Specifically, 61 strains, mainly distributed in the Group 2 and Group 5, were isolated from the potable water system in the International Space Station, with isolation dates ranging from 2009 to 2015. Five strains (5/76, 6.6%, mainly distributed in Group 3 and Group 4) were isolated from humans (*Homo sapiens*), suggesting potential pathogenicity. These findings indicate that two clusters of strains from the International Space Station may represent different initial contaminants from Earth, rather than two evolutionary paths of a single contaminant. However, both clusters were exposed to the same environmental conditions, specifically drinking water.

### Pan-genomic analysis revealed the genetic characteristics of *Ralstonia pickettii*

**Figure 2:**
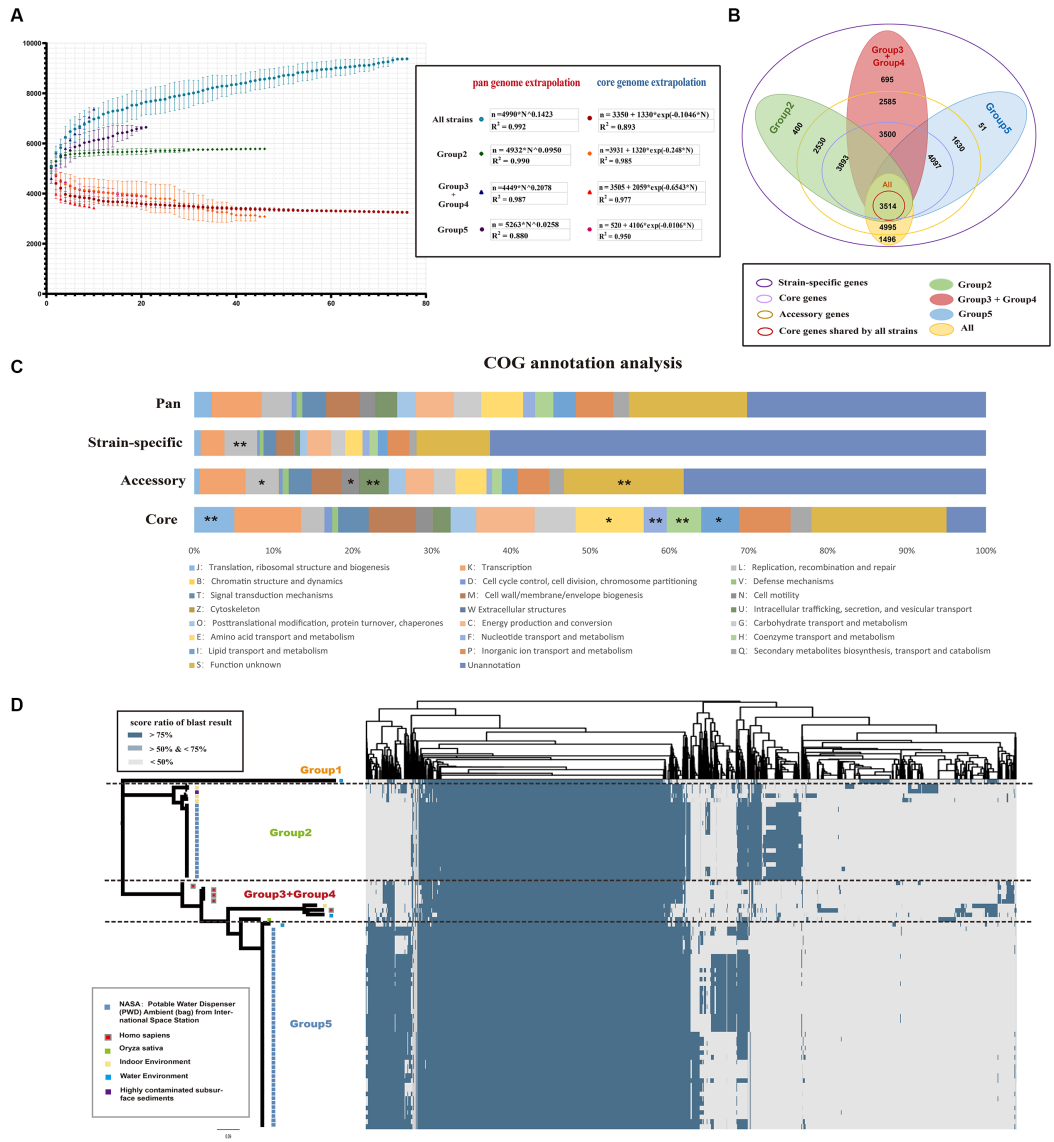
Core and pan-genomic analysis of *Ralstonia pickettii.* **(A)** Progressive curves were estimated for the core genome and pan-genome of all strains, as well as for different groups (Group 2, Group 3 + Group 4, and Group 5). These curves demonstrate a decrease in the number of core gene families and an increase in the number of pan-gene families as more genomes are added. Mathematical functions describing the pan-genome curves are displayed within the frame. **(B)** A Venn plot illustrates the gene content, including core genome, accessory genome, and strain-specific genes, among different groups of *Ralstonia pickettii* genomes. **(C)** The distribution of cluster of orthologous group (COG) categories is shown for the pan-genome, core genome, accessory genome, and strain-specific genes. Statistical significance is denoted by asterisks (^*^Fisher’s exact test *p*-value < 0.05; ^**^Fisher’s exact test *p*-value < 0.01). **(D)** A cluster heatmap presents the pan-genome of *Ralstonia pickettii*, showing the clustering patterns of gene families.

To further characterize the genetic characteristics of *Ralstonia pickettii*, pan-genome analysis was performed. A total of 10,005 pan-genome gene families were identified (Figure 2B). Among these, 3,514 (35.1%) represented the core-genome, and the remaining 6,491 (54.9%) represented the accessory genome (4,995, 49.9%) and strain-specific genes (1,496, 15.0%). Cluster of orthologous group (COG) annotation analysis was performed to categorize the function of pan-gene families. As shown in Figure 2C, the core-genome was significantly enriched in categories, such as COG-J (translation, ribosomal structure, and biogenesis), COG-E (amino acid transport and metabolism), COG-F (nucleotide transport and metabolism), COG-H (coenzyme transport and metabolism), and COG-I (lipid transport and metabolism) [COG-J, -E, and -I: Fisher’s exact test *p*-value < 0.05; COG-F, and -H: Fisher’s exact test *p*-value < 0.01]. These genes are primarily involved in maintaining normal physiological functions and material metabolism in bacteria. On the other hand, the accessory genes were enriched in categories, such as COG-L (replication, recombination, and repair), COG-N (cell motility), and COG-U (intracellular trafficking, secretion, and vesicular transport). These findings suggest that movement in different environments and interactions with various materials are necessary for the development of new traits.

The pan-genome accumulation curve, representing the increasing number of genomes, followed Heaps’ law (*n* = $кN^{\gamma }$ ) pan-genome model (Figure 2A), with γ = 0.1423. A positive exponent (γ > 0) indicated an open pan-genome, suggesting that novel accessory gene families that may be identified as additional strains are sampled. The pan-genome of Group 2, Group 3 + Group 4, and Group 5 also exhibited a linear upward trend (the strain number of Group 1 was too small and will not be discussed in this section). However, different power law values suggested different degrees of openness within each group. As shown in Figure 2A, the pan-genome of Group 3 + Group 4 had a higher exponent (γ = 0.2078), indicating a larger source gene pool and potential for adapting to new niches by acquiring novel genetic elements. In contrast, Group 2 (γ = 0.0950) and Group 5 (γ = 0.0258) experienced relatively simpler environmental pressures during evolution. The results of the pan-genome all-blast-all analysis are shown in Figure 2D (detailed information of pan-blast-screen results is shown in Supplementary Table S2), which is consistent with our population genetic analysis.

### Genetic plasticity and genomic evolution mediated by numerous MGEs and HGT

**Figure 3:**
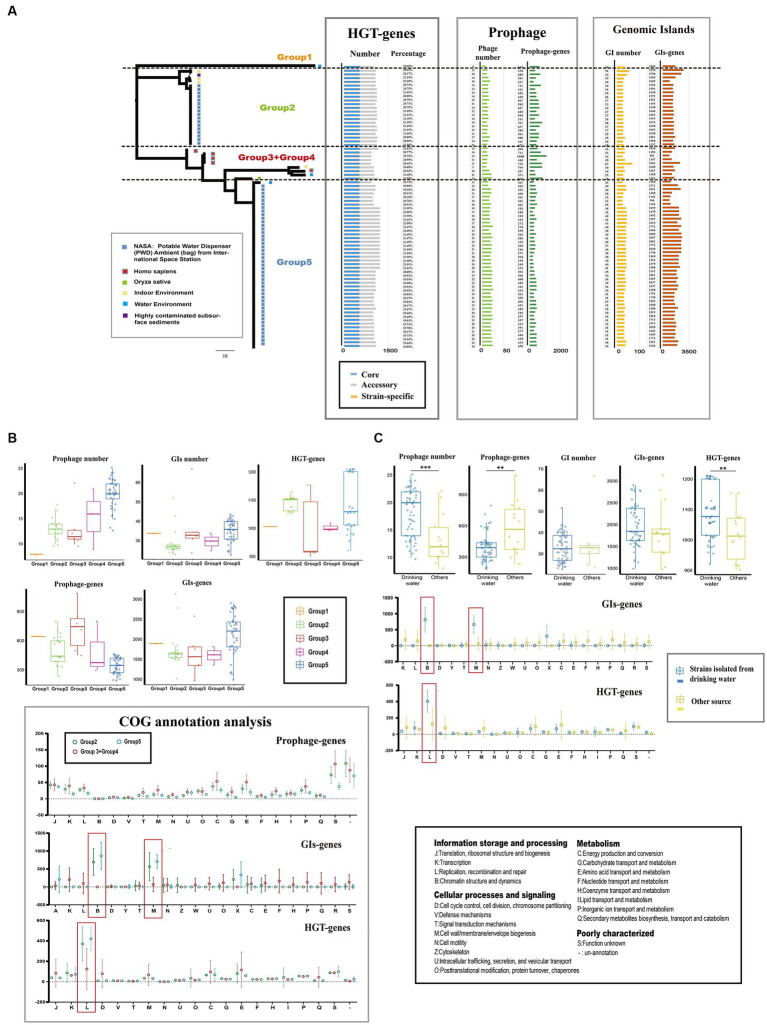
Mobile genetic elements (MGEs) and Horizontal gene transfers (HGTs) events in *Ralstonia pickettii*. **(A)** The distribution of mobile genetic elements (MGEs) and horizontally acquired genes in each strain is depicted. **(B)** The gene quantity and functional analysis of MGEs and horizontal gene transfer (HGT) events in different groups are presented. **(C)** A comparison is made between MGEs and horizontally acquired genes in strains isolated from drinking water and those obtained from other sources. Functional categories that show significant enrichment are highlighted with red boxes and were subjected to statistical testing (*t*-test). Statistical significance is indicated by asterisks (^**^t-test *p*-value < 0.01; ^***^t-test *p*-value < 0.001).

Mobile genetic elements (MGEs) and horizontal gene transfer (HGT) play crucial roles in genetic diversity and the expansion of gene pools of bacteria (Ochman et al., 2000; Gyles and Boerlin, 2014). In the case of *Ralstonia pickettii*, we analyzed the distribution of MGEs and HGT events (Figure 3A; Supplementary Table S3). On average, each genome contained 17.3 ± 8 genomic islands (GIs), 33.4 ± 1 prophages, and 1078.3 ± 34.5 HGT genes. There were significant differences in the number and gene content of prophages (prophage genes) among the different groups. Group 5 had the highest number of prophages (20.2 ± 2.8), while Group 2 had a lower number of prophages (13.1 ± 0.5). However, Group 5 had a lower gene content of prophages compared with the other groups. Similar variations were observed in the number and gene content of GIs (GI genes) and HGT genes (HGT genes) among the groups. The distribution of MGEs and HGT genes in *Ralstonia pickettii* contributes to the formation of genomic diversity during evolution. After COG annotation analysis (Figure 3B), we observed that Group 2 and Group 5 had relatively fewer prophage genes in almost every COG category. However, GI genes of Group 2 and Group 5 were significantly enriched in COG-B (chromatin structure and dynamics) and COG-M (cell wall/membrane/envelope biogenesis), while HGT genes of Group 2 and Group 5 were significantly enriched in COG-L (replication, recombination, and repair). These specific gene distributions are likely related to the environmental adaptation of *Ralstonia pickettii* in the drinking water of the International Space Station, where strains from Group 2 and Group 5 are predominantly found. Further investigation revealed that strains isolated from drinking water had a higher number of prophages but a smaller number of prophage genes compared with the other strains (Figure 3C). This indicates differences in prophage species. More HGT genes were observed in strains isolated from drinking water, while no significant differences were found in the number of GIs and GI genes. Consistent with the previous results, GI genes were significantly enriched in COG-B and COG-M, while HGT genes were significantly enriched in COG-L in *Ralstonia pickettii* isolated from drinking water. The diverse distribution of MGEs and HGT genes drive genetic plasticity and genomic evolution in *Ralstonia pickettii*, particularly in complex genetic backgrounds. Through these processes, *Ralstonia pickettii* can acquire novel metabolic properties to adapt to extreme environmental conditions.

### Comparative core-genomic and pan-genomic analysis between different groups

**Figure 4:**
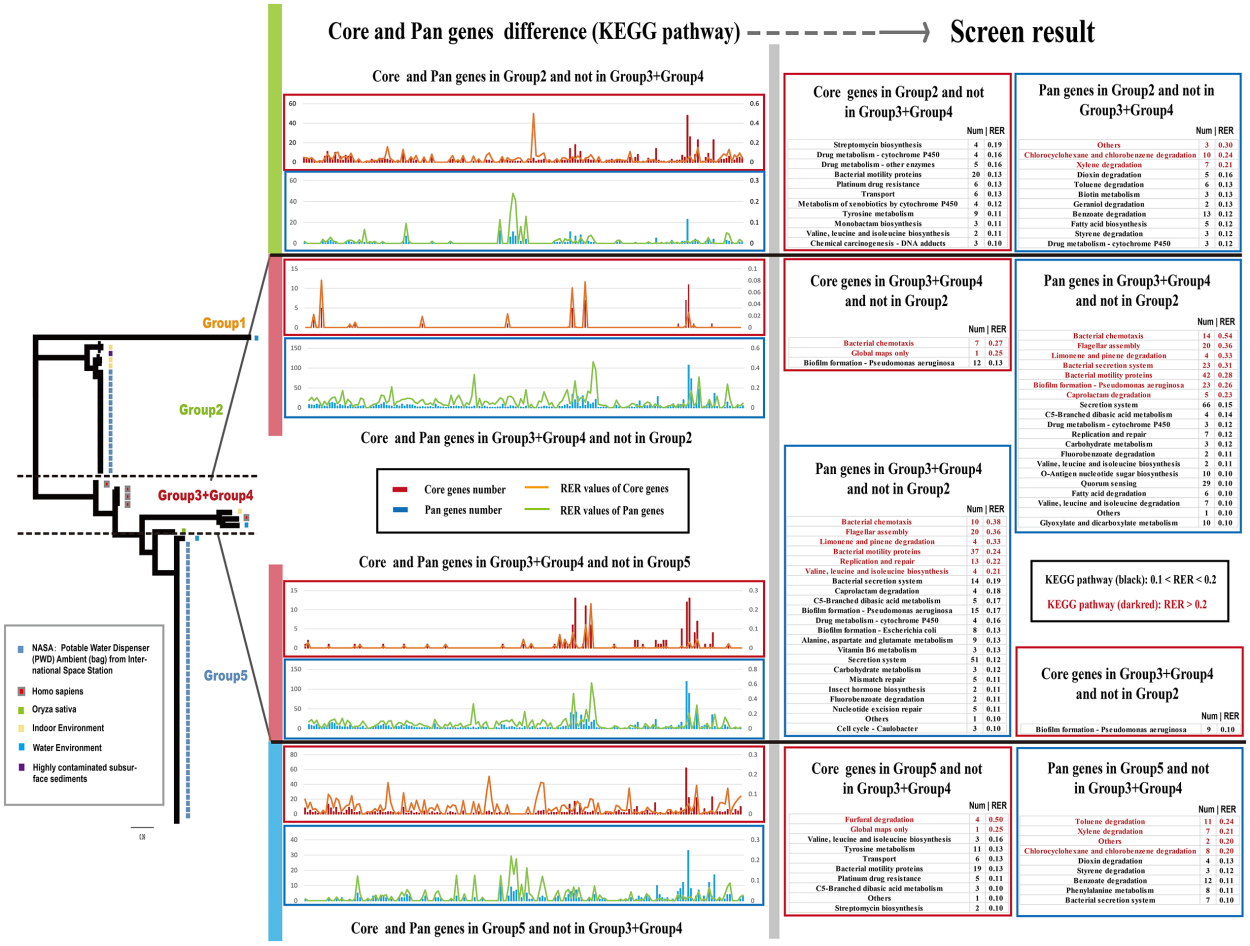
Screen results of comparative core- and pan-genomic analysis between different Groups. The chart displays a bar chart showing the number of genes and a line chart representing the Relative Enrichment Ratio (RER) for each KEGG pathway. The RER is a measure of the correlation between gene enrichment and the impact on the corresponding pathway. On the right side of the chart, the screen result of the comparative core- and pan-genomic analysis is presented. To provide visual clarity, KEGG pathways with RER values between 0.1 and 0.20 are labeled in black, while pathways with RER values greater than 0.20 are labeled in dark red.

The genetic characteristics observed in different groups of *Ralstonia pickettii* can be attributed to the development of unique core-genomes and pan-genomes during the adaptive evolutionary process under different genetic backgrounds. To understand the genomic characteristics of each group, a KEGG function enrichment analysis was conducted on the core-genomes and pan-genomes of each group (Supplementary Figure S1; Supplementary Table S9). The results were presented using two methods: gene function and pathway. However, no significant differences were found between the core-genomes and pan-genomes of each group. To compare the core-genomes and pan-genomes between different groups, a comparative analysis was performed focusing on specific KEGG pathways and genes associated with environmental signal response, critical substance synthesis, and metabolism (Figure 4). The bar charts in different colors represent the core-genome and pan-genome differences between Group 2 and Group 3 + Group 4, as well as Group 3 + Group 4 and Group 5 (Group 1, with a small number of strains, is not discussed in this section). To assess the impact of changes in gene numbers on each KEGG pathway, the relative enrichment ratio (RER) was calculated using RER values of 0.1 and 0.2 as screening criteria. The effective enrichment pathways from the comparative core-genomic and pan-genomic analysis between different groups are presented in Figure 4 (Supplementary Table S4). Although the pan-genome contains the core-genome, differences were observed between the core-genomes and pan-genomes of the two adjacent groups. This is because our analysis method removes the influence of the number of metabolic pathway background genes. In the pan-genome of Group 2 and not in Group 3 + Group 4, enriched pathways included others, chlorocyclohexane and chlorobenzene degradation, xylene degradation, and some metabolism-related pathways (especially disinfectant substances). On the other hand, pathways such as bacterial chemotaxis, flagellar assembly, bacterial secretion system, biofilm formation, replication and repair, and nutrient metabolism-related pathways were mainly enriched in the pan-genome of Group 3 + Group 4 and not in Group 2. In the core-genome, pathways such as bacterial chemotaxis and biofilm formation were enriched in Group 3 + Group 4 and not in Group 2, while pathways related to transport, drug metabolism, and some metabolism-related pathways were found in the core-genome of Group 2 and not in Group 3 + Group 4. In the pan-genome of Group 3 + Group 4 and not in Group 5, enriched pathways included bacterial chemotaxis, flagellar assembly, bacterial secretion system, biofilm formation, caprolactam degradation, limonene and pinene degradation, valine, leucine, and isoleucine biosynthesis, and nutrient metabolism-related pathways. Pathways such as furfural degradation, transport, and some metabolism-related pathways were enriched in the core-genome of Group 5 and not in Group 3 + Group 4. The pan-genome of Group 5 showed enrichment in pathways such as chlorocyclohexane and chlorobenzene degradation, others, and some metabolism-related pathways (especially disinfectant substances) that were not found in Group 3 + Group 4. These differences in core-genomes and pan-genomes between different groups reveal the genomic characteristics formed through the adaptive evolution of *Ralstonia pickettii* under different environmental conditions. Additionally, these results indicate the diversity of the *Ralstonia pickettii* genome.

### The key mutation accumulation of *Ralstonia pickettii* during the adaptive evolution from complex environmental conditions to drinking water

**Figure 5:**
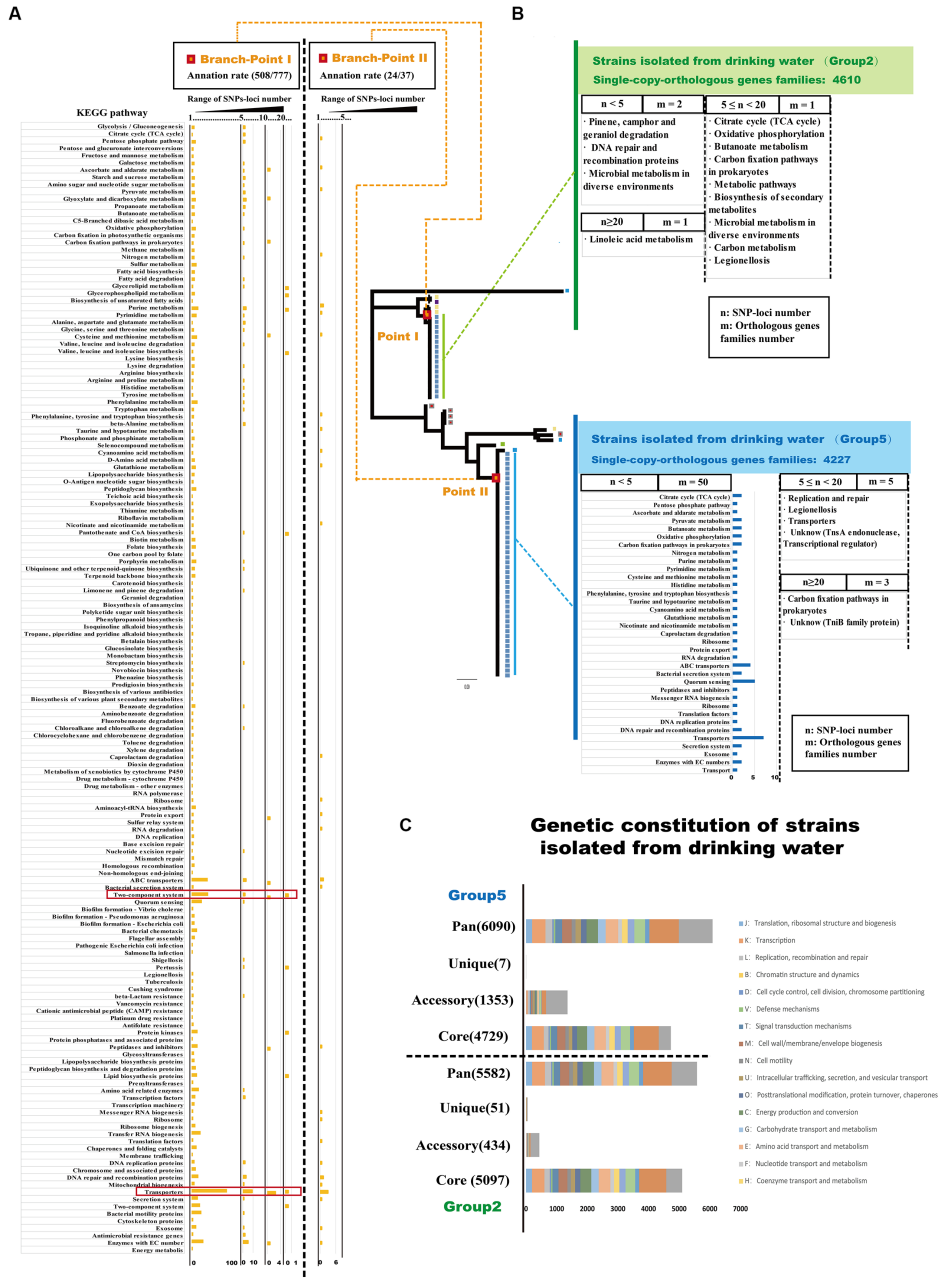
SNPs-loci distribution reveals the adaptive evolutionary characteristic of *Ralstonia pickettii* in drinking water. **(A)** The distribution of SNPs-loci at the two key branch-points of the phylogenetic tree is depicted. The bar chart represents the enrichment of orthologous gene families with SNPs-loci after KEGG annotation. The results are divided into three ranges based on the number of SNPs-loci (1 < *n* < 5, 5 ≤ *n* < 10, 10 ≤ *n* < 20, *n* ≥ 20), and the corresponding KEGG pathways are labeled on the left. **(B)** The distribution of SNPs-loci in *Ralstonia pickettii* strains isolated from drinking water is shown. The results are divided into three ranges based on the number of SNPs-loci (1 < *n* < 5, 5 ≤ *n* < 20, *n* ≥ 20), and different groups are color-coded (green and blue). The bar chart represents the function enrichment of orthologous gene families with SNPs-loci after KEGG annotation. **(C)** The genomic characteristics and COG annotation of strains isolated from drinking water in Group 2 and Group 5 are presented.

Specific gene mutations play a crucial role in promoting adaptive evolution in bacteria and can lead to genetic changes that help microorganisms adapt to diverse environmental conditions (Levin and Bergstrom, 2000; Chattopadhyay et al., 2013). To analyze the gene mutations associated with adaptive evolution in *Ralstonia pickettii* in drinking water, we examined the distribution of single nucleotide polymorphisms (SNPs) loci and their functional annotations in key branch-points on the phylogenetic tree (Figure 5A; Supplementary Table S5). As shown in Figure 5A, it can be observed that SNP loci occurred at branch-point I was associated with 777 orthologous gene families. Functional enrichment analysis based on KEGG annotation revealed several pathways that were affected by SNP loci. Orthologous gene families with a high number of SNP loci (*n* ≥ 20) were associated with pathways, such as glycerolipid metabolism, glycerophospholipid metabolism, valine, leucine, and isoleucine biosynthesis, pantothenate and CoA biosynthesis, two-component system, pertussis, protein kinases, and lipid biosynthesis proteins. Notably, there were 84 SNP loci found in orthologous gene families associated with purine metabolism. Orthologous gene families with a moderate number of SNP loci (10 ≤ *n* < 20) were linked to pathways, such as transporter, two-component system, peptidases and inhibitors, protein export, glycine, serine, and threonine metabolism, carbon fixation in photosynthetic organisms, pyruvate metabolism, galactose metabolism, and glycolysis/gluconeogenesis. Similarly, orthologous gene families with a lower number of SNP loci (5 ≤ *n* < 10) were also associated with pathways, such as transporter and two-component system. Even genes with fewer accumulated SNPs (1 < *n* < 5) were primarily distributed in pathways related to transporter, two-component system, quorum sensing, enzymes with EC numbers, and bacterial motility proteins. At branch-point II, SNP loci were associated with 37 orthologous gene families. The functional enrichment analysis based on KEGG annotation indicated that orthologous gene families with a lower number of SNP loci (1 < *n* < 5) were associated with the pathway transporters. The presence of SNP loci at specific branch-points in the phylogenetic tree suggests their role in shaping specific branches and clusters in population genetics analysis. Thus, the distribution of these SNP loci can provide insights into the accumulation of mutations during the adaptive evolution of *Ralstonia pickettii* under complex genetic backgrounds. These mutations are likely to affect specific physiological functions, providing the organism with the potential ability to survive in drinking water.

### Genetic characteristics of *Ralstonia pickettii* in the process of adaptive evolution in drinking water

Drinking water creates a unique environmental pressure on *Ralstonia pickettii*, leading to potential variations in its genetic characteristics during the process of adaptive evolution. To analyze these variations, we conducted a genomic analysis of strains isolated from drinking water in Group 2 and Group 5 by COG annotation (Figure 5C; Supplementary Table S6). As shown in Figure 5C, strains isolated from drinking water in the Group 2 exhibited a higher number of core orthologous gene families compared with the Group 5. However, strains from drinking water in the Group 5 displayed a larger number of accessory and unique orthologous gene families. These findings may be attributed to HGT events occurring between *Ralstonia pickettii* and other bacteria within the same environmental niche. It is worth noting that strains of *Burkholderia* have also been isolated from the potable water system in the International Space Station (O'Rourke et al., 2020), indicating the occurrence of HGT events in similar environments. Additionally, the strains isolated from drinking water in the Group 5 had a higher number of HGT genes compared with other strains in this study, further supporting the notion of genetic exchange through HGT. This aligns with the previous results, where strains from the Group 5 exhibited more HGT genes than other strains in this research. To analyze the gene mutations accumulated during the adaptive evolution of *Ralstonia pickettii* in drinking water, we examined the distribution of SNP loci in strains isolated from drinking water (Figure 5B). In the Group 2, SNP loci were identified in four orthologous gene families. Two orthologous gene families had a small number of SNP loci (1 < *n* < 5) and were associated with KEGG pathways, such as pinene, camphor, and geraniol degradation, DNA repair and recombination proteins, and microbial metabolism in diverse environments. One orthologous gene family had a moderate number of SNP loci (5 ≤ *n* < 10) and was involved in various KEGG pathways, including the citrate cycle (TCA cycle), oxidative phosphorylation, butanoate metabolism, carbon fixation pathways in prokaryotes, biosynthesis of secondary metabolites, microbial metabolism in diverse environments, carbon metabolism, and legionellosis. One orthologous gene family had a high number of SNP loci (*n* ≥ 20) and was linked to the KEGG pathway, such as linoleic acid metabolism. In the Group 5, SNP loci were distributed across 58 orthologous gene families. Fifty orthologous gene families had a small number of SNP loci (1 < *n* < 5) and were mainly associated with carbohydrate metabolism, energy metabolism, transporters, and quorum sensing. Five orthologous gene families had a moderate number of SNP loci (5 ≤ *n* < 10) and were involved in KEGG pathways, such as replication and repair, legionellosis, transporters, and an unknown pathway (TnsA endonuclease, transcriptional regulator). Three orthologous gene families had a high number of SNP loci (*n* ≥ 20) and were associated with KEGG pathways, including carbon fixation pathways in prokaryotes and an unknown pathway (TniB family protein: recombinase). Overall, the analysis suggests that under the environmental pressure of drinking water, *Ralstonia pickettii* accumulates mutations in orthologous gene families associated with linoleic acid metabolism, carbon fixation pathways in prokaryotes, and DNA recombination and repair. Notably, many orthologous gene families that accumulate mutations are related to carbon fixation pathways associated with the energy metabolism of *Ralstonia pickettii*.

### Potential pathogenicity and drug resistance of *Ralstonia pickettii*

**Figure 6:**
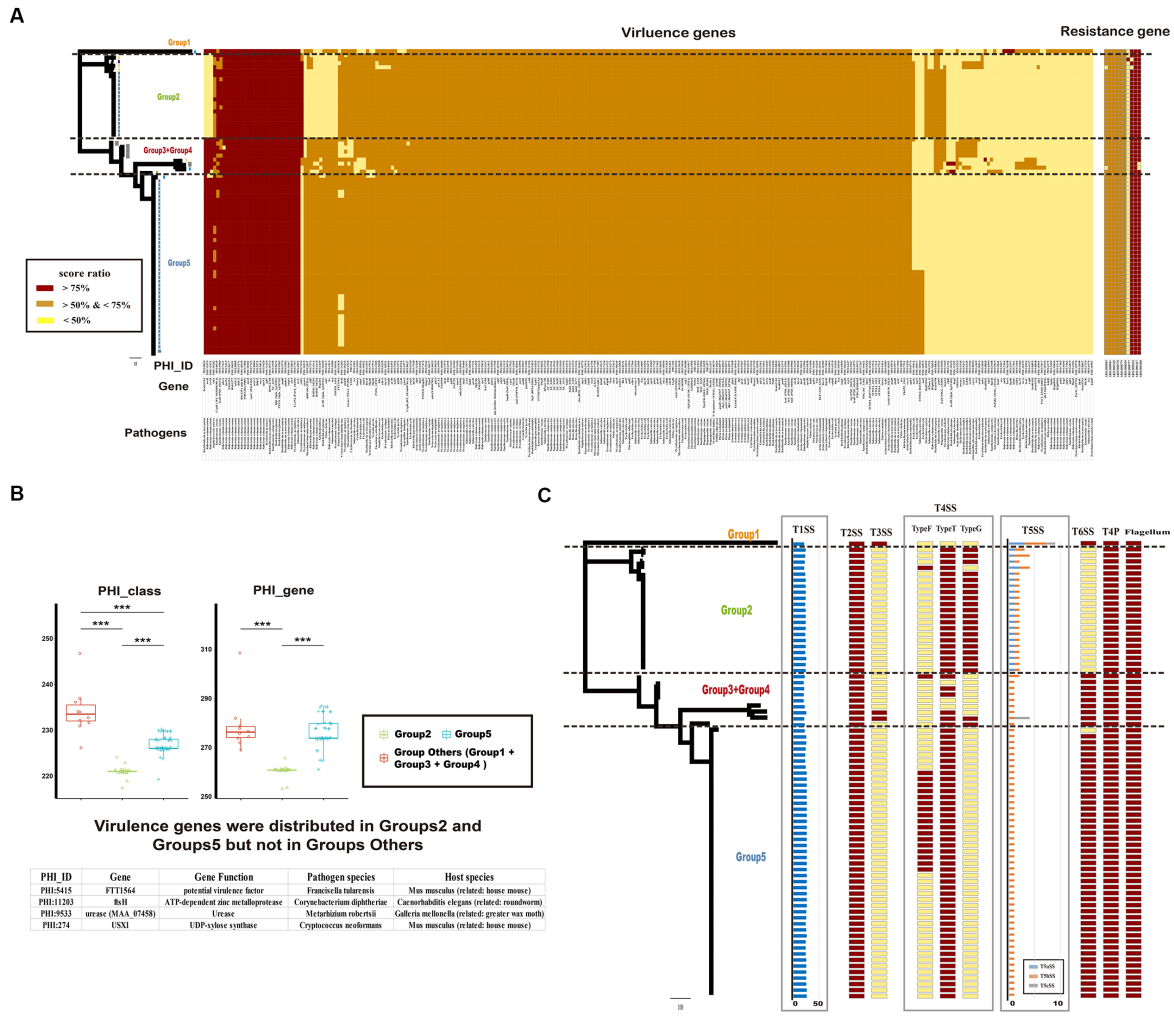
Potential pathogenicity and drug resistance of *Ralstonia pickettii* strains. **(A)** The heatmap displays the distribution of virulence genes based on the PHI database and resistance genes based on the CARD database. Each gene is color-coded based on the score ratio from the blast result of each genome. The PHI-ID, gene name, and associated pathogen are labeled below the heatmap. **(B)** The box chart shows the difference in virulence genes among different groups (Group 2, Group 5, and Group Others: Group 1 + Group 3 + Group 4). The chart indicates the number of virulence genes and PHI classes for each group. Statistical significance is indicated by asterisks (^***^t-test *p*-value < 0.001). Virulence genes that are exclusively present in Group 2 and Group 5 are listed at the bottom. **(C)** The distribution of macromolecular secretion systems in *Ralstonia pickettii* is represented. Dark red boxes indicate the presence of a macromolecular system within a genome, while yellow boxes indicate the absence of a macromolecular system.

To identify potential pathogenic characteristics, the virulence genes of *Ralstonia pickettii* were investigated. A total of 285 gene families were identified that matched with virulence genes in the PHI database (Figure 6A and the detail information is shown in Supplementary Table S7). Among these gene families, 213 (74.7%) were shared by almost all *Ralstonia pickettii* strains, indicating a potential shared pathogenic capacity. Additionally, it was found that all *Ralstonia pickettii* strains possess the general secretory pathway (GSP) from *Ralstonia solanacearum*. The study also examined the role of virulence genes in the adaptive evolution of *Ralstonia pickettii* in drinking water. It was observed that strains in Group 1 + Group 3 + Group 4 had a significantly higher number of virulence genes (234.2 ± 10.5) compared with other groups. In contrast, strains of the Group 2 had fewer virulence genes (262.8 ± 24.0) and PHI classes (222.1 ± 13.0) compared with the other groups (Figure 6B). Specific virulence genes mainly distributed in the Groups 2 and 5 were identified, including genes associated with potential virulence factors from other bacteria. These genes may be related to the environmental adaptability of *Ralstonia pickettii* in drinking water.

Furthermore, the presence of macromolecular secretion systems, such as type I (T1SS), type II (T2SS), type III (T3SS), type IV (T4SS), type V (T5SS), and type VI (T6SS) secretion systems, as well as type IV pilus (T4P), flagellum, and Tad pilus, was investigated (Figure 6C; Supplementary Table S8). It was found that all *Ralstonia pickettii* strains possessed T1SS, T2SS, T4P, and flagellum. T3SS was present only in certain strains of Group 1 and Group 3 + 4, indicating a relatively strong virulence in these groups. Different types of T4SS and T5SS were also identified in *Ralstonia pickettii* strains, with variations in their distribution among different groups. Additionally, we examined the genotypic profiles of antimicrobial resistance in *Ralstonia pickettii*. It was found that almost all strains possessed eight antimicrobial resistance genes: ARO:3003049 (*rosB*), ARO:3003010 (*ceoB*), ARO:3000778 (*adeG*), ARO:3000779 (*adeH*), ARO:3001417 (*OXA-22*), ARO:3001808 (*OXA-60*), ARO:3000501 (*rpoB2*), and ARO:3003105 (*dfrA3*), which was associated with resistance-nodulation-cell division (RND) antibiotic efflux pump, major facilitator superfamily (MFS) antibiotic efflux pump, beta-lactamase, and trimethoprim-resistant dihydrofolate reductase. These resistance genes may confer corresponding clinical resistance phenotypes to *Ralstonia pickettii*. Overall, the analysis of virulence genes, macromolecular secretion systems, and antimicrobial resistance genes provides insights into the potential pathogenic characteristics and adaptive evolution of *Ralstonia pickettii* in drinking water environments.

## Discussion

*Ralstonia pickettii,* being a versatile pathogen, can be found in various habitats such as water conditions, sediments, plants, and drinking water. As a generalist, *Ralstonia pickettii* likely maintains large genomes with a higher number of functional genes to adapt to diverse environmental conditions (Thomas et al., 2016). The presence of an open pan-genome with variable gene families plays a crucial role in the genome plasticity and genetic diversity of *Ralstonia pickettii*, which is significant for its adaptive evolution (Pandit et al., 2009; Sriswasdi et al., 2017). The analysis conducted in this study revealed the potential role of both the core-genome and accessory genes in the adaptive evolution of *Ralstonia pickettii*. The core genome, which consists of genes shared by all strains, was found to be enriched in categories related to substance synthesis, nutrient transport, and metabolism (COG-J, −E, -F, -H, and -I). These essential genes enable *Ralstonia pickettii* to efficiently acquire nutrients from the environment and adapt metabolically to occupy different ecological niches (Goyal, 2018; Iranzo et al., 2019; Cummins et al., 2022). On the other hand, the accessory genome, which consists of genes present in some but not all strains, was enriched in categories associated with bacterial proliferation, motility, and substance transport (COG-L, -N, and -U). These genes contribute to maintaining normal physiological functions in bacteria and provide an evolutionary dynamic to keep the pan-genome open under different environmental stresses (Garcia-Garcera and Rocha, 2020; Maistrenko et al., 2020).

Phylogenetic and genetic population analyses have divided *Ralstonia pickettii* into five distinct groups. Strains isolated from drinking water in the International Space Station (ISS) were primarily distributed in the Group 2 and Group 5, while strains associated with humans (*Homo sapiens*) were found in the Group 3 and Group 4. Comparative analysis of the core- and pan-genomes among these groups has revealed the diverse genomic characteristics of *Ralstonia pickettii* during its adaptive evolution under different environmental conditions. The pan-genome of strains in the Group 2 or Group 5 exhibited a high abundance of genes associated with the metabolism of specific substances, particularly those involved in the degradation of common disinfectants. Pathways such as chlorocyclohexane and chlorobenzene degradation, xylene degradation, dioxin degradation, toluene degradation, and benzoate degradation were prominently represented. Additionally, the core-genome of strains in the Group 2 or Group 5 contained genes associated with pathways related to transport, drug metabolism, and certain amino acid metabolism. However, these genes were not found in the Group 3 + Group 4 strains. Importantly, it is worth noting that most strains in the Group 2 and Group 5 were isolated from the potable water system in the International Space Station, where current physical and chemical methods are generally effective at maintaining water cleanliness. Bacteria residing in the potable water system are constantly exposed to extreme environmental pressures from drugs, disinfectants, and oligotrophic conditions (Wong et al., 2010; Bornemann et al., 2015; Thornhill and Kumar, 2018). The unique core- and pan-genomes found in strains from the Group 2 and Group 5 likely contribute to the environmental adaptability of *Ralstonia pickettii*, enabling its survival in the potable water system of the International Space Station. On the other hand, the pan-genome and core-genome specific to strains in the Group 3 + Group 4 were primarily associated with pathways such as bacterial chemotaxis, flagellar assembly, bacterial secretion system, biofilm formation, and nutrient metabolism-related pathways. These genes potentially provide *Ralstonia pickettii* with enhanced motility, stress resistance, and the ability to respond to and utilize diverse nutrients, thereby achieving better environmental adaptability under complex environmental conditions (Tolker-Nielsen, 2015; Goyal, 2018; Rossi et al., 2018; Colin et al., 2021). Furthermore, the differences in the pan-genomic composition among different groups highlight the close relationship between prokaryotic non-core genes and various evolutionary models of population structure and dynamics. Gene composition evolution through gene gain and loss tends to occur at a faster rate than sequence evolution (Iranzo et al., 2019).

Genetic drift and gene conversion are crucial processes in the adaptive evolution of bacteria, contributing to genetic plasticity and genomic changes (Power et al., 2021). Our analysis revealed significant differences in the number of genomic islands (GIs), prophages, and horizontally transferred genes (HGT) among the different groups (Figure 3B), indicating the role of genetic plasticity and genomic evolution driven by mobile genetic elements and HGT in *Ralstonia pickettii*. The functional differences observed in GI genes and HGT genes among strains in different groups suggest that these processes introduce new characteristics during bacterial adaptation (Rosenberg, 2001). Specifically, we observed that GI genes in the Group 2 and Group 5 were significantly enriched in COG-B and COG-M functional categories, while HGT genes in these groups were significantly enriched in COG-L. Similar functional enrichments were observed when comparing GI genes and HGT genes between strains isolated from drinking water and other environments. The low osmotic pressure in drinking water imposes greater environmental stress on bacteria, necessitating the presence of genes involved in envelope stress response. Additionally, gene families associated with DNA replication, recombination, and repair (COG-L) likely play crucial roles in the adaptive plasticity of the bacterial genome by allowing individuals to adjust their recombination and mutation rates (Poolman et al., 2002; Pandit et al., 2009; Paul, 2013; Milner et al., 2023). Our study demonstrates that *Ralstonia pickettii* may acquire important genes through GIs and HGT to achieve environmental adaptation in water environments.

During adaptive evolution, bacteria undergo mutations in key orthologous gene families, leading to the emergence of new branches in phylogenetic trees (Wellenreuther et al., 2019; Wei et al., 2022). In our study, we focused on two branch-points associated with strains isolated from drinking water. We observed that orthologous gene families associated with pathways such as transporters and two-component systems were more prone to accumulating mutations and forming single nucleotide polymorphisms (SNPs). Transporter proteins are considered ecological assets and features of microbial pangenomes, capable of providing niche-defining phenotypes (Wellenreuther et al., 2019; Wei et al., 2022; Milner et al., 2023). Furthermore, during adaptive evolution in drinking water, *Ralstonia pickettii* accumulated specific gene mutations in key orthologous gene families related to linoleic acid metabolism, carbon fixation pathways in prokaryotes, and DNA recombination and repair. Notably, many of the orthologous gene families showing mutations were associated with carbon fixation pathways, indicating that the carbon metabolism and energy metabolism of *Ralstonia pickettii* are significantly influenced by environmental pressures during adaptive evolution in drinking water. These mutations are likely to impact related physiological functions and provide insights into the direction of adaptive evolution. On the other hand, microgravity also serves as an environmental factor for bacteria to live in the ISS. It has been reported that microgravity can enhance certain physiological functions of bacteria, such as growth, biofilm formation, virulence, and antibiotic resistance. However, most of these physiological changes are related to the regulation of gene expression (Singh et al., 2018; Vaishampayan et al., 2018; Vaishampayan and Grohmann, 2019). The potential impact of microgravity on this process can be discussed in future studies.

The virulence genotypic profiles and the distribution of macromolecular secretion systems revealed potential pathogenic characteristics of bacteria (Abby et al., 2014; Abby and Rocha, 2017; Urban et al., 2020). Most virulence genes (74.7%) are shared by all *Ralstonia pickettii* strains, indicating a potential shared pathogenicity. We focused on the virulence genes only primarily distributed in the Group 2 and Group 5, which may be related to the environmental adaptability of bacteria in drinking water. Based on the sequence alignment results, these genes associated with FTT1564 (potential virulence factor from *Francisella tularensis*), FtsH (ATP-dependent zinc metalloprotease *Corynebacterium diphtheria*), MAA_07458 (urease from *Metarhizium robertsii*), and USX1 (UDP-xylose synthase from *Cryptococcus neoformans*). The role of these virulence genes in the environmental adaptability of *Ralstonia pickettii* in drinking water needs further study. Macromolecular secretion systems, including T4SS, three types of T5SS (T5aSS, T5bSS, and T5cSS), and T6SS, reflected differences in potential pathogenicity among the different groups which were found in *Ralstonia pickettii*. The unique distribution of T5SS may significantly impact the potential virulence of different groups (Fan et al., 2016), but it may not contribute to environmental adaptation. T6SS has been reported to contribute to bacterial pathogenesis by translocating substrates in the host and facilitating competition with other bacteria in their niches (Grohmann et al., 2018). In *Ralstonia pickettii*, T6SS was found in all groups except the Group 2. The specific distribution of different macromolecular secretion systems among *Ralstonia pickettii* genomes suggests their potential pathogenicity under diverse environmental conditions. This suggests that these macromolecular secretion systems play a role in the pathogenicity of the *Ralstonia pickettii*. In addition, the antimicrobial resistance genotypic profiles of *Ralstonia pickettii* revealed the presence of eight antimicrobial resistance genes and were shared with almost all strains. OXA-60 and OXA-22 were two chromosomal resistance genes in *Ralstonia pickettii* (Nordmann et al., 2000; Girlich et al., 2004). Resistance-nodulation-cell division (RND) family has been reported as the dominant intrinsic and acquired multidrug resistance mechanism in *Burkholderia* species (Podnecky et al., 2015). The results indicated that these resistance genes, as the core gene, did not change with the evolution of the *Ralstonia pickettii* genome, and contributed to the clinical resistance phenotype, which highlighted the importance of addressing antimicrobial resistance in *Ralstonia pickettii* infections.

In conclusion, the comprehensive genomic analysis has provided valuable insights into the genetic diversity, potential pathogenicity, and adaptive evolution of *Ralstonia pickettii*. The study has highlighted the significance of virulence-related elements and antimicrobial resistance genes in the pathogenicity of bacteria. Moreover, the analysis has provided a deeper understanding of the genetic characteristics and adaptive evolutionary processes of *Ralstonia pickettii* in the context of the drinking water environment. These findings contribute to our knowledge of this bacterium and have implications for addressing its pathogenic potential and antimicrobial resistance in healthcare and environmental settings. Further research in this area will continue to enhance our understanding of *Ralstonia pickettii* and its interactions with its environment.

## Data availability statement

The datasets presented in this study can be found in online repositories. The names of the repository/repositories and accession number(s) can be found in the article/Supplementary material.

## Author contributions

CY: Conceptualization, Formal analysis, Funding acquisition, Investigation, Methodology, Software, Validation, Visualization, Writing – original draft, Writing – review & editing. TA: Data curation, Resources, Software, Writing – original draft. XL: Data curation, Resources, Software, Writing – review & editing. JZ: Data curation, Resources, Software, Writing – review & editing. ZL: Data curation, Resources, Software, Writing – review & editing. JG: Data curation, Resources, Software, Writing – review & editing. RH: Data curation, Investigation, Writing - review & editing. ZF: Conceptualization, Funding acquisition, Project administration, Supervision, Writing – review & editing.

## Footnotes

^1^ https://galaxy.pasteur.fr/

## Supplementary material

SUPPLEMENTARY FIGURE S1 Comparative core- and pan-genomic analysis between different Groups based on KEGG annotation. The histogram represented the gene-number of KEGG categories and the color of each Group corresponded to the color in Figure 1. Detailed information on the KEGG categories was shown on the bottom.

SUPPLEMENTARY TABLE S1 Detailed strain-information and genetic characteristics of the strains in this study.

SUPPLEMENTARY TABLE S2 Detailed information of pan-blast-screen results.

SUPPLEMENTARY TABLE S3 Detailed information of MGEs and HGT genes.

SUPPLEMENTARY TABLE S4 Screen and statistics results of comparative core-and pan-genome of each Group after KEGG annotation analysis.

SUPPLEMENTARY TABLE S5 Detailed information of the distribution of SNPs-loci at key branch-point.

SUPPLEMENTARY TABLE S6 Detailed information of mutation accumulation during adaption evolution in drinking water.

SUPPLEMENTARY TABLE S7 Detailed information of PHI-screen-results and corresponding PHI genes.

SUPPLEMENTARY TABLE S8 Detailed information of CARD-screen-results and the distribution of macromolecular secretion systems.

SUPPLEMENTARY TABLE S9 Screen and statistics results of core-and pan-genome of each Group after KEGG annotation analysis.
